# Effects of sex, tobacco smoking, and alcohol consumption osteoporosis development: Evidence from Taiwan biobank participants

**DOI:** 10.18332/tid/136419

**Published:** 2021-06-17

**Authors:** Chung-Yuan Yang, Jerry Cheng-Yen Lai, Wei-Lun Huang, Chiao-Lin Hsu, Shaw-Ji Chen

**Affiliations:** 1 Department of Psychiatry, Taitung MacKay Memorial Hospital, Taitung, Taiwan; 2 Department of Medical Research, Taitung MacKay Memorial Hospital, Taitung, Taiwan; 3 National Taitung University, Taitung, Taiwan; 4 Division of Family Medicine, Taipei Veterans General Hospital, Taitung Branch, Taitung, Taiwan; 5 Health Management Center, Kaohsiung Veterans General Hospital, Kaohsiung, Taiwan; 6 Kaohsiung Medical University, Kaohsiung, Taiwan; 7 Department of Medicine, MacKay Medical College, New Taipei City, Taiwan

**Keywords:** tobacco, alcohol, sex differences, osteoporosis, Taiwan Biobank

## Abstract

**INTRODUCTION:**

Osteoporosis is major public health concern, but the long-term impacts of tobacco and alcohol consumption on its development are unclear. This study analyzed the relationship between tobacco and alcohol use and osteoporosis by using data from the Taiwan Biobank (TWB), established in 2012.

**METHODS:**

Participants in TWB were included in our study, with a total of 18394 respondents included for analysis. To investigate the relationship between tobacco and alcohol use and osteoporosis, we surveyed their bone mineral density (BMD), consumption of tobacco and alcohol and other covariate data.

**RESULTS:**

We found that participants in the tobacco smoking only group (OR=1.24; 95% CI: 1.08–1.42, p=0.003) and the group that both smoked and consumed alcohol (OR=1.39; 95% CI: 1.09–1.77, p=0.008) were more likely to develop osteoporosis than were participants who neither drank alcohol nor smoked. Menopause is strongly associated with osteoporosis in women, and we found that women who used alcohol or tobacco were not at a significantly higher risk than those in the reference group (tobacco only, OR=1.15; 95% CI: 0.86–1.53, p=0.345; both tobacco and alcohol, OR=0.61; 95% CI: 0.14–2.60, p=0.5040). However, men in these groups were at a significantly higher risk than the reference group (tobacco only, OR=1.26; 95% CI: 1.07–1.48, p=0.006; both tobacco and alcohol, OR=1.32; 95% CI: 1.03–1.70, p=0.030). Menopause was a significant risk factor for osteoporosis (OR=2.46; 95% CI: 1.77–3.41, p<0.001).

**CONCLUSIONS:**

The influence of tobacco use on osteoporosis was significant, particularly in men, but the effects of alcohol consumption were equivocal in our study. The interactions between tobacco use, alcohol use, and menopausal status on osteoporosis should be considered in future studies.

## INTRODUCTION

Osteoporosis is an important risk factor affecting public health. Osteoporosis begins without major discomfort but leads to age-related fractures later in life^1^. Osteoporosis is a reduction in bone mineral density (BMD). Usually, it is asymptomatic and is therefore undetected until a fracture^2^. In people with osteoporosis, their bone tissue is qualitatively normal but their BMD is quantitatively reduced^3^. People with osteoporosis have a BMD lower than the average BMD of healthy adults of the sex and age group by 2.5 standard deviations (SD: T-score ≤ -2.5)^4^. The occurrence of osteoporosis is increasing in older adults, especially postmenopausal older women^5^. Osteoporosis is also a frequent cause of bone fractures. The commonest fracture sites among older adults are the hip, spine, and forearm, and such fractures have other osteoporosis-related effects (e.g. hospitalization, illness, and increased healthcare costs)^6^.

Complex interactions between genetic and environmental factors have been reported to contribute to the pathophysiology of osteoporosis^7-9^. These factors include tobacco smoking, alcohol consumption, low physical activity, weight change, nutrition intake and absorption (specifically, calcium and vitamin D), fracture history, corticosteroid use, hormonal factors, genetic factors, and female sex^9,10^.

Certain factors contributing to osteoporosis, such as sex and genetics, are not modifiable, but tobacco and alcohol use can be altered. However, the effects of tobacco and alcohol use on the development of osteoporosis are not well understood. Possible mechanisms of the effect of tobacco smoking on bone health remain unclear, and some findings are controversial^11^. Research indicates that tobacco smoking may cause an imbalance in the process of bone turnover, leading to lower bone mass and BMD, thus increasing susceptibility to osteoporosis^12^. Alcohol consumption is also associated with an increased incidence of fractures and complications^13^. However, the details of the complex effects of alcohol on bone tissue are not well understood. One study indicated that menopausal women who resumed alcohol use had reduced bone turnover markers^14^. Therefore, further research into the long-term effects of tobacco and alcohol consumption on osteoporosis is recommended.

In Taiwan, owing to its well-established healthcare system, life expectancy and therefore the proportion of older adults has been increasing. The 2005–2008 National Nutrition and Health Survey in Taiwan (NAHSIT) investigated the prevalence of osteoporosis by measuring BMD, and it was discovered that the prevalence of osteoporosis had increased since the 1999–2000 NAHSIT^15,16^. Tobacco smoking and alcohol consumption also increased in prevalence after 2000, when Taiwan became a member of WTO. Therefore, clarifying the effects of tobacco and alcohol consumption on osteoporosis is critical.

The aim of this study was to explore the influence of tobacco smoking and alcohol consumption on the development of osteoporosis. Data collected in the Taiwan Biobank (TWB) since 2012 could be used to identify possible interactions among tobacco smoking, alcohol consumption, and osteoporosis.

## METHODS

### The Taiwan Biobank (TWB)

The TWB is sponsored by the Taiwanese Government. Its purpose is to collect lifestyle and genetic data from Taiwanese people^17,18^. TWB is a populationbased dataset, which aims to recruit 200000 community-based healthy participants aged 30–70 years without history of cancer before 2024. In 2020, 29 recruitment centers contributed information on 105387 volunteering participants to the TWB. In addition to blood samples and physical examination, each participant completed a structured questionnaire on personal information and lifestyle factors through face-to-face interviews with a TWB researcher.

### BMD measurement

Broadband ultrasound attenuation (BUA) with highspeed quantitative ultrasound measurement was used to measure BMD by the TWB^13,19^. BUA is easy to administer and is therefore suitable for TWB data collection.

### Alcohol consumption and tobacco smoking

Alcohol consumption among participants was categorized by defining alcohol consumers as those with a weekly intake of over 150 cc of alcohol for at least 6 months at the time of BMD measurement. Tobacco smoking was also surveyed in this study. Tobacco smokers were those who had smoked regularly for at least 6 months by the time of their BMD measurement.

### Covariates

Covariate data were also collected (sex, age, body mass index [BMI], regularity of exercise, educational level, family history of osteoporosis, and diabetes mellitus [DM] status) for statistical analysis in accordance with previous studies^20-22^. In particular, gathering data on menopause was important in women, so only female participants aged ≥40 years were enrolled in the study. The demographic data were collected during the TWB enrollment interview. For the TWB, the body height and weight of each participant were measured, and BMI was calculated as weight (kg)/ (height in m)^2^.

### Statistical analysis

The characteristics between participants with different alcohol drinking and tobacco smoking behaviors were described by means and standard deviations for continuous and percentages for categorical data. Tea and drinking behaviors of participants were classified into one of four categories: no drinking and no smoking, only smoking but no drinking, only drinking but no smoking, or both drinking and smoking. All variables in the different groups were compared using one-way analysis of variance (ANOVA) for continuous data, and χ^2^ or Fisher’s exact tests for categorical data. Multivariable logistic regression was performed to estimate the adjusted odds ratios (AOR) for the risk of osteoporosis in men and women with tea and alcohol drinking behaviors, with adjustment for baseline demographic characteristics and lifestyle behavior. To account for gender as potential interaction associated with tea and drinking behaviors, separate multivariate logistic models were estimated for male and female participants. Independent variables included for adjustment in the multivariable logistic model was guided by the results from participant characteristics with p<0.05. All data transformations and statistical analyses were conducted by the SAS statistical software for Windows (Version 9.4; SAS Institute, Cary, NC, USA). The null hypothesis was rejected at an alpha level of 0.05.

## RESULTS

### Basic characteristics of TWB participants

After the exclusion of respondents aged <40 years (n=3819) and those with incomplete or missing information on independent variables of interest (n=189), 18394 participants were included for analysis. All details including sex, age, BMI, regular exercise, educational level, and DM are given in Table 1.

**Table 1:** Baseline characteristics of total participants stratified by alcohol and tobacco consumption behaviors in Taiwanese men and women aged 40–70 years (2008–2015) (N=18394)

*Characteristics*	*Neither alcohol nor tobacco (N=13733; 74.7%)*	*Tobacco but not alcohol (N=3621; 19.7%)*	*Alcohol but not tobacco (N=272; 1.5%)*	*Both alcohol and tobacco (N=768; 4.2%)*	
	*n (%)*	*n (%)*	*n (%)*	*n (%)*	*p*
**Age** (years), mean ± SD	54.8 ± 7.7	54.5 ± 7.9	53.8 ± 8.0	53.4 ± 7.7	<0.001***
**Male**	2529 (18.4)	2962 (81.8)	153 (56.3)	726 (94.5)	<0.001***
**Residential area**					
Rural	833 (6.1)	258 (7.1)	23 (8.5)	75 (9.8)	<0.001***
Non-Rural	12900 (93.9)	3363 (92.9)	249 (91.5)	693 (90.2)	
**Education level**					
College or graduate school	8205 (59.7)	1901 (52.5)	168 (61.8)	456 (59.4)	<0.001***
High school, elementary school, or less	5528 (40.3)	1720 (47.5)	104 (38.2)	312 (40.6)	
**Baseline comorbidity**					
Family history of osteoporosis	2092 (15.2)	537 (14.8)	45 (16.5)	109 (14.2)	0.725
Diabetes mellitus	790 (5.8)	300 (8.3)	13 (4.8)	56 (7.3)	<0.001***
BMI, mean ± SD	23.8 ± 3.4	25.0 ± 3.4	24.6 ± 3.3	25.4 ± 3.2	<0.001***
**BMI** (kg/m^2^)					
<25	9335 (68.0)	1938 (53.5)	160 (58.8)	349 (45.4)	<0.001***
≥25	4398 (32.0)	1683 (46.5)	112 (41.2)	419 (54.6)	
**BMD profile**					
T-Score, mean ± SD	-0.6 ± 1.6	-0.9 ± 1.5	-0.5 ± 1.6	-0.9 ± 1.4	<0.001***
**T-score** ≤ **-2.5 (Outcome)**					
No	11955 (87.1)	3157 (87.2)	241 (88.6)	658 (85.7)	0.588
Yes	1778 (12.9)	464 (12.8)	31 (11.4)	110 (14.3)	
**Behavioral factors**					
Regular exercise	7202 (52.4)	1733 (47.9)	154 (56.6)	374 (48.7)	<0.001***

SD: standard deviation. BMD: bone mineral density.

*** p<0.001; χ2 test or one-way analysis of variance test. Percentages refer to each column total N.

All participants were classified into four groups according to their smoking and drinking status, including: ‘neither alcohol nor tobacco’ (n=13733; 74.7%); ‘tobacco but not alcohol’ (n=3621; 19.7%), ‘alcohol but not tobacco’ (n=272; 1.5%), ‘both alcohol and tobacco’ (n=768; 4.2%); the average ages (SD) were, respectively, 54.8 (7.7), 54.5 (7.9), 53.8 (8.0), and 53.4 (7.7) years in the four groups (Table 1). The numbers and percentages of men were 2529 (18.4%), 2962 (81.8%), 153 (56.3%), and 726 (94.5%) in the four groups, respectively. The numbers and percentages of participants living in rural areas were 833 (6.1%), 258 (7.1%), 23 (8.5%) and 75 (9.8%) in the four groups, respectively. The numbers and percentages of participants with a college degree were 8205 (59.7%), 1901 (52.5%), 168 (61.8%), and 456 (59.4%) in the four groups, respectively. The numbers and percentages of participants with DM were 790 (5.8%), 300 (8.3%), 13 (4.8%), and 56 (7.3%) in the four groups, respectively. The numbers and percentages of participants with a BMI ≥25 were 4398 (32.0%), 1683 (46.5%), 112 (41.2%), and 419 (54.6%) in the four groups, respectively. The numbers and percentages of participants who exercised regularly were 7202 (52.4%), 1733 (47.9%), 154 (56.6%), and 374 (48.7%) in the four groups, respectively. These covariates were all (p<0.001) significantly different between the four groups (Table 1).

### Influence of tobacco and alcohol on osteoporosis

Participants who did no use alcohol or tobacco were the reference group and were compared with participants in the other three groups. A T-score of ≤2.5 for BMD was considered to indicate osteoporosis. We selected all variables that were determined to be significant (p<0.05) during the univariate analysis as covariates in the logistic regression analysis. Participants who smoked only or used both tobacco and alcohol were more likely to have osteoporosis compared with the reference group (AOR=1.24; 95% CI: 1.08–1.42 and AOR=1.39; 95% CI: 1.09–1.77; respectively). Participants were less likely to have osteoporosis in other categories, such as those who had a college degree (AOR=0.86; 95% CI: 0.78–0.96), who had DM (AOR=0.78; 95% CI: 0.65–0.93), who had a BMI of <25 (AOR=0.70; 95% CI: 0.64–0.78), and who regularly exercised (AOR=0.84; 95% CI: 0.77–0.92) (Table 2).

**Table 2:** The effect of alcohol and tobacco consumption behaviors on the risk of osteoporosis in Taiwanese men and women aged 40–70 years (2008–2015)

*Independent variables*	*AOR (95% CI)*	*p*
**Drinking and smoking behavior** (vs neither alcohol nor tobacco)		
Tobacco but not alcohol	1.24 (1.08–1.42)	0.003**
Alcohol but not tobacco	1.04 (0.69–1.56)	0.854
Both alcohol and tobacco	1.39 (1.09–1.77)	0.008**
**Age** (years)	1.08 (1.08–1.09)	<0.001***
**Female** (vs male)	0.90 (0.80–1.03)	0.120
**Residential area** (rural vs non-rural)	1.05 (0.86–1.29)	0.603
**Education level** (vs none or elementary school, high school)		
College or graduate school	0.86 (0.78–0.96)	0.005**
**Baseline comorbidity**		
Diabetes mellitus (vs no)	0.74 (0.61–0.89)	0.002**
BMI (vs BMI <25)	0.70 (0.63–0.78)	<0.001***
**Behavioral factors**		
Regular exercise (vs no)	0.78 (0.71–0.86)	<0.001***

BMI: body mass index (kg/m^2^). CI: confidence interval. AOR: adjusted odds ratio.

### Influence of sex, tobacco use, and alcohol consumption on osteoporosis

We also investigated the differences in the effects of alcohol consumption and tobacco smoking in women and men by performing a subgroup analysis. Due to the possible influence of menopause on osteoporosis in women, we included menopause as a covariate in the logistic regression analysis. The risk of osteoporosis in men was still significant in those who only smoked (AOR=1.26; 95% CI: 1.07–1.48) and those who used both alcohol and smoked (AOR=1.32; 95% CI: 1.03– 1.70). Women did not have a significantly higher risk of osteoporosis if they only smoked (AOR=1.15; 95% CI: 0.86–1.53) or if they used both alcohol and smoked (AOR=0.61; 95% CI: 0.14–2.60). Menopause was a risk factor for osteoporosis (AOR=2.46; 95% CI: 1.77–3.41). Age was the only risk factor across women and men (AOR=1.10; 95% CI: 1.09–1.11 and AOR=1.04; 95% CI: 1.03–1.05, respectively). Two protective factors identified were a BMI of <25 for both women and men (AOR=0.59; 95% CI: 0.52–0.68 and AOR=0.81; 95% CI: 0.70–0.93, respectively) and regular exercise for both women and men (AOR=0.75; 95% CI: 0.66–0.85 and AOR=0.80; 95% CI: 0.69–0.94, respectively). Women with a college degree had a lower risk of osteoporosis but men with a college degree did not (AOR=0.86; 95% CI: 0.75–0.98 and AOR=0.91; 95% CI: 0.78–1.06, respectively). The influence of DM in different genders was equivocal (AOR=0.86; 95% CI: 0.75–0.98 and AOR=0.91; 95% CI: 0.78–1.06, respectively) (Table 3).

**Table 3:** Effects of alcohol consumption and tobacco smoking on the risk of developing osteoporosis in men and women aged 40–70 years (2008–2015)

*Independent variables*	*Male*		*Female*	
	*AOR (95% CI)*	*p*	*AOR (95% CI)*	*p*
**Drinking and smoking behavior** (vs neither alcohol nor tobacco)				
Tobacco but not alcohol	1.26 (1.07–1.48)	0.006**	1.15 (0.86–1.53)	0.345
Alcohol but not tobacco	0.84 (0.47–1.47)	0.537	1.38 (0.77–2.47)	0.277
Both alcohol and tobacco	1.32 (1.03–1.70)	0.030*	0.61 (0.14–2.60)	0.504
**Age** (years)	1.04 (1.03–1.05)	<0.001***	1.10 (1.09–1.11)	<0.001***
**Menopausal status** (vs pre-menopausal)				
Post-menopausal			2.46 (1.77–3.41)	<0.001***
**Residential area** (rural vs non-rural)	1.10 (0.82–1.47)	0.529	0.99 (0.75–1.31)	0.950
**Education level** (vs none or elementary school, high school)				
College or graduate school	0.91 (0.78–1.06)	0.221	0.86 (0.75–0.98)	0.025*
**Baseline comorbidity**				
Diabetes mellitus (vs no)	0.69 (0.52–0.92)	0.012*	0.82 (0.63–1.06)	0.124
BMI (vs BMI <25)	0.81 (0.69–0.94)	0.006**	0.58 (0.51–0.67)	<0.001***
**Behavioral factors**				
Regular exercise (vs no)	0.80 (0.69–0.94)	0.007**	0.75 (0.66–0.85)	<0.001***

BMI: body mass index (kg/m^2^). CI: confidence interval. AOR: adjusted odds ratio.

### Analysis of the alcohol only group

The osteoporosis risk in the alcohol consumption only group was not significantly higher than in the reference group; however, subgroups of women and men had significantly higher and lower risks of osteoporosis, respectively. We found an AOR of 1.04 (95% CI: 0.69–1.56) for this group, but within the subgroup of women (AOR=1.38; 95% CI: 0.77–2.47) and men (AOR=0.84; 95% CI: 0.47–1.47) significant differences were observed. The percentages of women (43.8%) and men (56.3%) were almost equal in the alcohol consumption only group, and the overall percentage of men was higher than that in the nondrinking and non-smoking group (18.4%) but lower than that in the smoking-only group (81.8%) and in the both smoking and drinking group (94.5%). The baseline characteristics of the alcohol consumption only group stratified by sex are shown in Table 4. The percentage of women with a college degree (77.3%) was significantly higher than the percentage of men (49.7%) in this group (p<0.001) The percentages of men with a family history of osteoporosis, DM, or a BMI >25 (20.9%, 7.2%, and 52.3%, respectively) were also significantly higher than those of women with these conditions (10.9%, 1.7%, and 26.9%, respectively) in this group.

**Table 4:** Baseline characteristics of men and women who consumed alcohol but did not smoke aged 40–70 years (2008–2015) (N=272)

*Independent variables*	*Male (n=153; 56.3%) n (%)*	*Female (n=119; 43.8%) n (%)*	*p*
**Age** (years), mean ± SD	54.5 ± 8.4	52.7 ± 7.3	0.063
**Residential area**			
Rural	15 (9.8)	8 (6.7)	0.365
Non-Rural	138 (90.2)	111 (93.3)	
**Education level**			
College or graduate school	76 (49.7)	92 (77.3)	<0.001***
High school, elementary school, or less	77 (50.3)	27 (22.7)	
**Baseline comorbidity**			
Family history of osteoporosis	32 (20.9)	13 (10.9)	0.028*
Diabetes mellitus	11 (7.2)	2 (1.7)	0.035*
BMI, mean ± SD	25.5 ± 3.0	23.5 ± 3.3	<0.001***
BMI			
<25	73 (47.7)	87 (73.1)	<0.001***
≥25	80 (52.3)	32 (26.9)	
**BMD profile**			
T-Score, mean ± SD	-0.7 ± 1.4	-0.4 ± 1.7	0.168
**T-score** ≤ **-2.5 (Outcome)**			
No	135 (88.2)	106 (89.1)	0.829
Yes	18 (11.8)	13 (10.9)	
**Behavioral factors**			
Regular exercise	86 (56.2)	68 (57.1)	0.878

BMI: body mass index (kg/m^2^). SD: standard deviation. BMD: bone mineral density.

*** p<0.001; χ2 test or two-sample t-test.

## DISCUSSION

We investigated the influence of tobacco smoking and alcohol consumption on the development of osteoporosis by analyzing the data collected by the TWB because these influences were unclear in previous research^23-26^.

First, we found the participants in the smokingonly group and both smoking and drinking group to be at a higher risk of developing osteoporosis than those in the non-smoking and non-drinking groups. This finding is important for confirming the risk of tobacco smoking in developing osteoporosis, and it is statistically powerful due to the large sample size provided by the TWB. Second, we separated men and women for further analysis because menopause is strongly associated with osteoporosis in women^27^. We found that men who smoked, regardless of alcohol consumption status, had a significantly higher risk of developing osteoporosis than the reference group, although no such risk was found among women with the same characteristics. Therefore, analyses of the influence of tobacco and alcohol on osteoporosis should be performed for participants grouped by sex.

Third, covariates other than tobacco and alcohol consumption were used to adjust the statistical analysis. Covariates such as regular exercise and a BMI >25 were determined to be protective factors in our study, in agreement with previous studies^28,29^. Age, another covariate in our study, was also determined to be a risk factor for osteoporosis, as in previous research^30^. However, DM was not correlated with a reduced risk of developing osteoporosis in our study, a finding inconsistent with those of some previous studies^31,32^. To investigate this inconsistency, we separated TWB enrollees into subgroups of men and women and performed an additional analysis. DM significantly increased the risk of developing osteoporosis in men but not in women; this constitutes additional evidence of sex differences in osteoporosis development.

The most significant difference between middleaged men and women was menopause. Menopause was the most significant co-existing risk factor for osteoporosis in our study. Osteoporosis after menopause with the loss of ovarian function and estrogen deficiency is a complex process involving numerous pathways and cytokines in the regulation of osteoclastogenesis^33^.

Postmenopausal osteoporosis might be prevented by adequate estrogen supplementation. The confounding effect of menopause status could explain why the effects of tobacco smoking and alcohol consumption on osteoporosis risk were non-significant in women.

### Limitations

Our study had limitations. First, this study was crosssectional; a longitudinal study might be more effective in evaluating the influences of tobacco and alcohol on osteoporosis over time. Second, daily consumption of tobacco and alcohol were not easy to calculate in the study due to the design of the TWB questionnaire. Further cohort studies could better assess the longterm effects of alcohol and tobacco use, particularly if quantitative data on daily alcohol and tobacco use are collected as part of the study protocol.

## CONCLUSIONS

The influence of tobacco use on osteoporosis development was significant, but the effects of alcohol use were unclear. Specifically, men who use tobacco are at a higher risk of osteoporosis development. However, menopausal status was also a major risk factor for osteoporosis development in women. The interactions between tobacco, alcohol, and menopause on osteoporosis should be considered in the further studies.

## Data Availability

The data supporting this research is available from the authors on reasonable request.
